# Development of comprehensive nomograms for evaluating overall and cancer-specific survival of laryngeal squamous cell carcinoma patients treated with neck dissection

**DOI:** 10.18632/oncotarget.15414

**Published:** 2017-02-16

**Authors:** Xiao Shi, Wei-ping Hu, Qing-hai Ji

**Affiliations:** ^1^ Department of Head and Neck Surgery, Fudan University Shanghai Cancer Center, Shanghai, China; ^2^ Department of Oncology, Shanghai Medical College, Fudan University, Shanghai, China; ^3^ Department of Respiratory Medicine, Zhongshan Hospital, Fudan University, Shanghai, China

**Keywords:** laryngeal squamous cell carcinoma, nomogram, overall survival, cancer-specific survival, lymph node ratio

## Abstract

**Background:**

Neck dissection for laryngeal squamous cell carcinoma (LSCC) patients could provide complementary prognostic information for AJCC N staging, like lymph node ratio (LNR). The aim of this study was to develop effective nomograms to better predict survival for LSCC patients treated with neck dissection.

**Results:**

2752 patients were identified and randomly divided into training (*n* = 2477) and validation (*n* = 275) cohorts. The 3- and 5-year probabilities of cancer-specific mortality (CSM) were 30.1% and 37.2% while 3- and 5-year death resulting from other causes (DROC) rate were 6.2% and 11.3%, respectively. 13 significant prognostic factors including LNR for overall (OS) and 12 (except race) for CSS were enrolled in the nomograms. Concordance index as a commonly used indicator of predictive performance, showed the nomograms had superiority over the no-LNR models and TNM classification (Training-cohort: OS: 0.713 vs 0.703 vs 0.667, CSS: 0.725 vs 0.713 vs 0.688; Validation-cohort: OS: 0.704 vs 0.690 vs 0.658, cancer-specific survival (CSS): 0.709 vs 0.693 vs 0.672). All calibration plots revealed good agreement between nomogram prediction and actual survival.

**Materials and Methods:**

We identified LSCC patients undergoing neck dissection diagnosed between 1988 and 2008 from Surveillance, Epidemiology, and End Results (SEER) database. Optimal cutoff points were determined by X-tile program. Cumulative incidence function was used to analyze cancer-specific mortality (CSM) and death resulting from other causes (DROC). Significant predictive factors were used to establish nomograms estimating overall (OS) and cancer-specific survival (CSS). The nomograms were bootstrapped validated both internally and externally.

**Conclusions:**

Comprehensive nomograms were constructed to predict OS and CSS for LSCC patients treated with neck dissection more accurately.

## INTRODUCTION

In the United States, there are estimated 13430 new cases diagnosed with laryngeal cancer in 2016 and laryngeal squamous cell carcinoma (LSCC) accounts for the vast majority of all laryngeal malignancies [1, 2]. In patients with LSCC, particularly for supraglottic area with abundant lymphatic network, there is a relatively high incidence of macro- or micrometastases, and involvement of even one lymph node might lead to a 50% reduction in survival time [3, 4]. Currently it’s hard to detect these micrometastases by non-invasive methods [5, 6]. As a result, NCCN guideline recommends neck dissection for LSCC patients who are at risk for occult lymph node metastases in order to examine and remove the potential nodal focus [7]. Besides, neck dissection could also determine the necessity of adjuvant therapy and provide important predictive and prognostic information like lymph node ratio (LNR), which was defined as the number of pathologically positive nodes divided by the total counts of examined lymph nodes.

**Figure 1:**
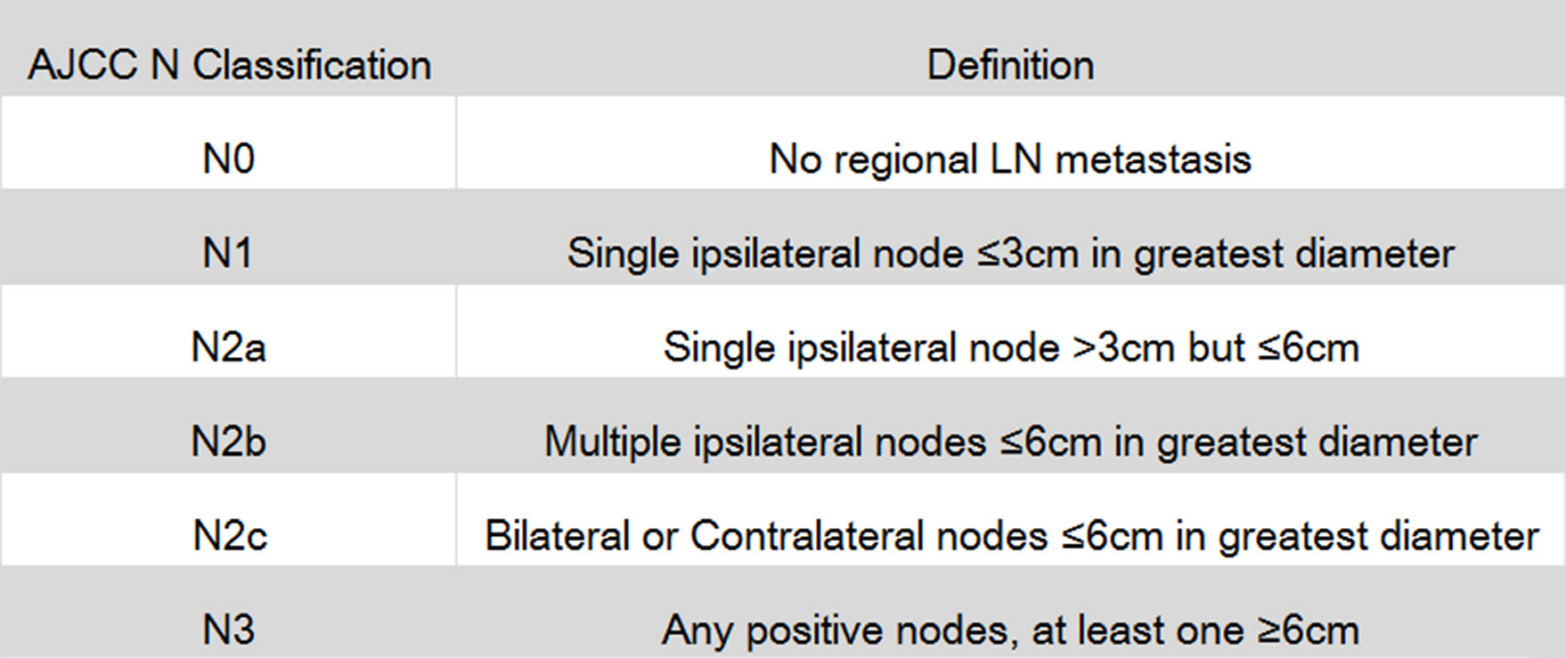
American Joint Committee on Cancer (AJCC) N staging for laryngeal cancer LN is short for lymph node.

Integrating information on regionally metastatic burden with the extent of neck dissection, prognostic value of LNR for LSCC patients has been verified by several previous studies with large sample size [8–11]. However, AJCC N staging only provides information on maximal size, number and laterality of metastatic lymph nodes (Figure 1). Therefore, LNR can be used as a supplementary factor for N staging to describe lymph node status. In addition, AJCC T classification for laryngeal cancer was merely based on range of tumor invasion, combination of T staging and tumor size is also likely to better reflect tumor status from different aspects.

There is a growing trend to use nomogram for cancer prognosis, specific nomograms have been successfully built for estimating survival, recurrence or local control for many cancer types [12–15]. Egelmeer et al have constructed nomograms for laryngeal cancer patients, however, the patients enrolled in that study only included patients treated with radiotherapy alone and whether those patients underwent neck dissection was unclear [16]. To the best of our knowledge, for LSCC patients, especially for those who underwent neck dissection, nomograms which make full use of available prognostic factors to predict survival have not been reported yet. Therefore in this study, we aimed to develop more practical and effective models for estimating OS and CSS for LSCC patients treated with neck dissection, in order to assist clinicians in predicting LSCC patient’s individualized survival.

## RESULTS

### Patient baseline characteristics

A total of 2752 LSCC patients treated with neck dissection diagnosed between 1988 and 2008 were identified from Surveillance, Epidemiology, and End Results (SEER) database. We randomly allocated 2477 patients into the training cohort and other 275 in the validation cohort.

For the training cohort, the median follow-up time until censoring or death was 58 months (Range: 1–311 months) and the median age was 60 years old (Range: 24–96). Of these 2477 patients in the training cohort, 79.7% were male and 73.5% were white. We also observed that primary tumor in 57.3% of patients who received neck dissection arose from supraglottis, 53% were staged N0 while more than 60% were staged T3 and T4. Besides of neck dissection, 33.1% of enrolled patients received only cancer-directed surgery (30.3%) or only radiotherapy (2.8%) while the others (66.9%) underwent both of the treatment modalities. (Note: information on chemotherapy was not accessible in SEER database.) Results of neck dissection showed that the median of node examination counts was 25 (Range: 1–89) and the median of LNR was 0.019 (Range: 0–1), respectively. By the cutoff date of follow-up, 1159 patients (46.8%) had died from primary cancer and 612 (28.7%) died from other causes (Table 1).

**Table 1:** Baseline demographics and clinical characteristics of patients

Characteristic	All patients		Training cohort		Validation cohort	
	*n* = 2752		*n* = 2477		*n* = 275	
	No.	%	No.	%	No.	%
**Categorical variables**						
Gender						
Female	569	20.7	503	20.3	66	24.0
Male	2183	79.3	1974	79.7	209	76.0
**Race**						
White	2019	73.4	1820	73.5	199	72.4
Black	617	22.4	547	22.1	70	25.5
Other^1^	116	4.2	110	4.4	6	2.2
**Marital status**						
Married	1380	50.1	1254	50.6	126	45.8
Unmarried^2^	1372	49.9	1223	49.4	149	54.2
**Grade**						
Well differentiated	257	9.3	237	9.6	20	7.3
Moderately differentiated	1568	57.0	1426	57.6	142	51.6
Poorly differentiated	888	32.3	794	32.1	94	34.2
Undifferentiated	39	1.4	20	0.8	19	6.9
**Site**						
Glottis	855	31.1	773	31.2	82	29.8
Supraglottis	1576	57.3	1418	57.2	158	57.5
Subglottis	93	3.4	84	3.4	9	3.3
Overlapping lesion	228	8.3	202	8.2	26	9.5
**Cancer-directed surgery**						
No cancer-directed surgery	85	3.1	70	2.8	15	5.5
Local excision/destruction^3^	459	16.7	403	16.3	56	20.4
Total/Radical laryngectomy	2208	80.2	2004	80.9	204	74.2
**Radiotherapy**						
No radiotherapy	838	30.5	751	30.3	87	31.6
Receive radiotherapy	1914	69.5	1726	69.7	188	68.4
**AJCC T status**						
T1	322	11.7	288	11.6	34	12.4
T2	755	27.4	700	28.3	55	20.0
T3	404	14.7	346	14.0	58	21.1
T4a	1175	42.7	1049	42.3	126	45.8
T4b	98	3.6	94	3.8	4	1.5
**AJCC N status**						
N0	1300	47.2	1165	47.0	135	49.1
N1	420	15.3	372	15.0	48	17.5
N2a	119	4.3	108	4.4	11	4.0
N2b	573	20.8	534	21.6	39	14.2
N2c	292	10.6	255	10.3	37	13.5
N3	48	1.7	43	1.7	5	1.8
**AJCC M status**						
M0	2691	97.8	2424	97.9	267	97.1
M1	61	2.2	53	2.1	8	2.9
**Continuous variables**						
**Age at diagnosis**						
Median (Range)	60 (24–96)		60 (24–96)		60 (25–85)	
**Tumor size (cm)**						
Median (Range)	3.0 (0.1–23.0)		3.0 (0.1–23.0)		3.1 (0.2–10.0)	
**Number of LN examined**						
Median (Range)	25 (1–89)		25 (1–89)		27 (1–89)	
**Number of positive LNs**						
Median (Range)	1 (0–58)		1 (0–58)		1 (0–17)	
**Lymph node ratio**						
Median (Range)	0.019 (0–1)		0.019 (0–1)		0.017 (0–1)	

^1^Including American Indian/Alaska Native, Asian/Pacific Islander.

^2^Including patients who are never married, divorced, separated or widowed.

^3^Local destruction includes photodynamic therapy, electrocautery, cryosurgery, laser ablation, etc.

SEER 1988–2008.

Clinicopathologic characteristics of the validation cohort were also listed in Table 1. Follow-up time ranged from 1 to 189 months (Median: 50) and 205 (74.5%) patients had died before the last follow-up, in which 132 (48.0%) were due to cancer.

### Optimal cutoff values of LNR and Tumor size

In order to construct nomograms, we need to stratify the continuous variables in Table 1 into several categories. X-tile program, a practical tool for cut-point optimization, was used to determine the optimal cutoff values for tumor size and LNR by the minimal *P*-value approach [17].

**Figure 2:**
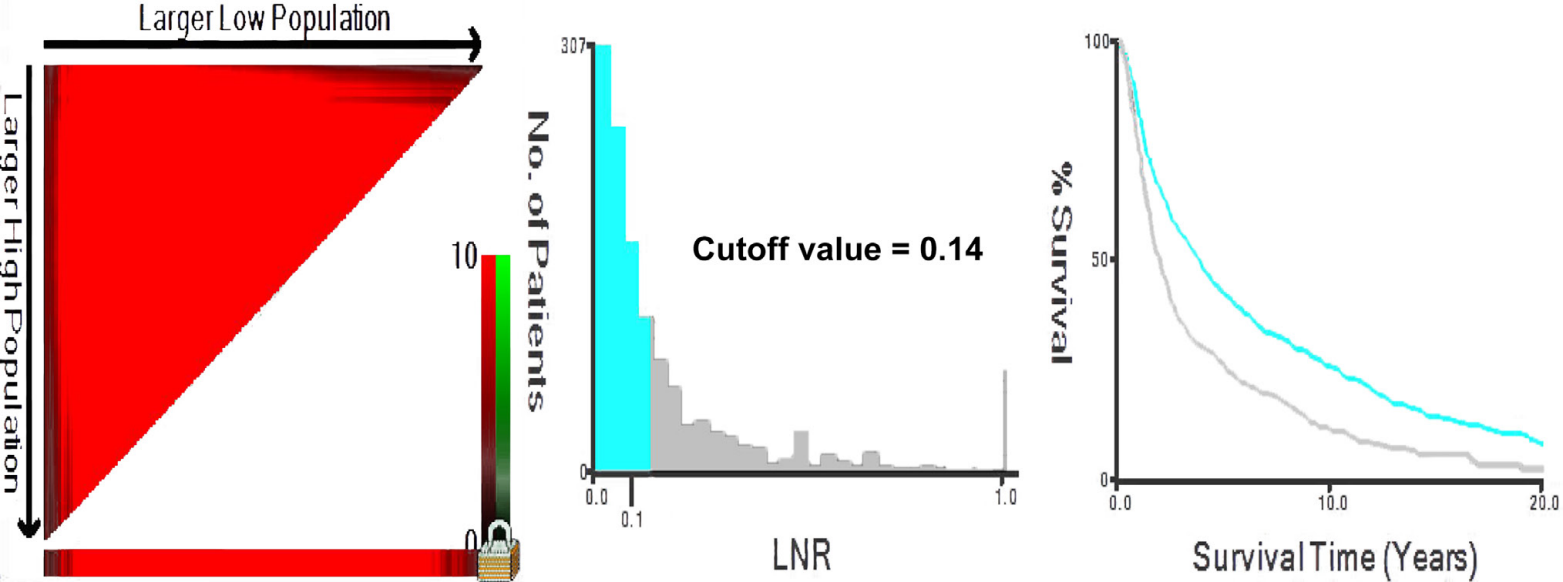
X-tile analysis identifying optimal LNR cutoffs based on OS X-tile analysis was conducted on patients with positive lymph nodes in the training cohort (*n* = 1312), these 1312 patients in the training cohort was equally divided into training (*n* = 656) and validation sets (*n* = 656). X-tile plots of training sets are shown in the left panels, the “lock” symbol in the left panel means optimal cutoffs have been determined, a histogram (middle panels) and a Kaplan-Meier plot (right panels) was performed based on these cutoffs. *P* values were determined by using the cut-point defined in the training set and applying it to the validation set. Optimal LNR cut-point was determined as 0.14 based on OS (χ2 = 55.675, *P <* 0.001). As the X axis could only show one decimal place, so we added a text annotation “Cutoff value = 0.14” in the middle panel.

**Figure 3:**
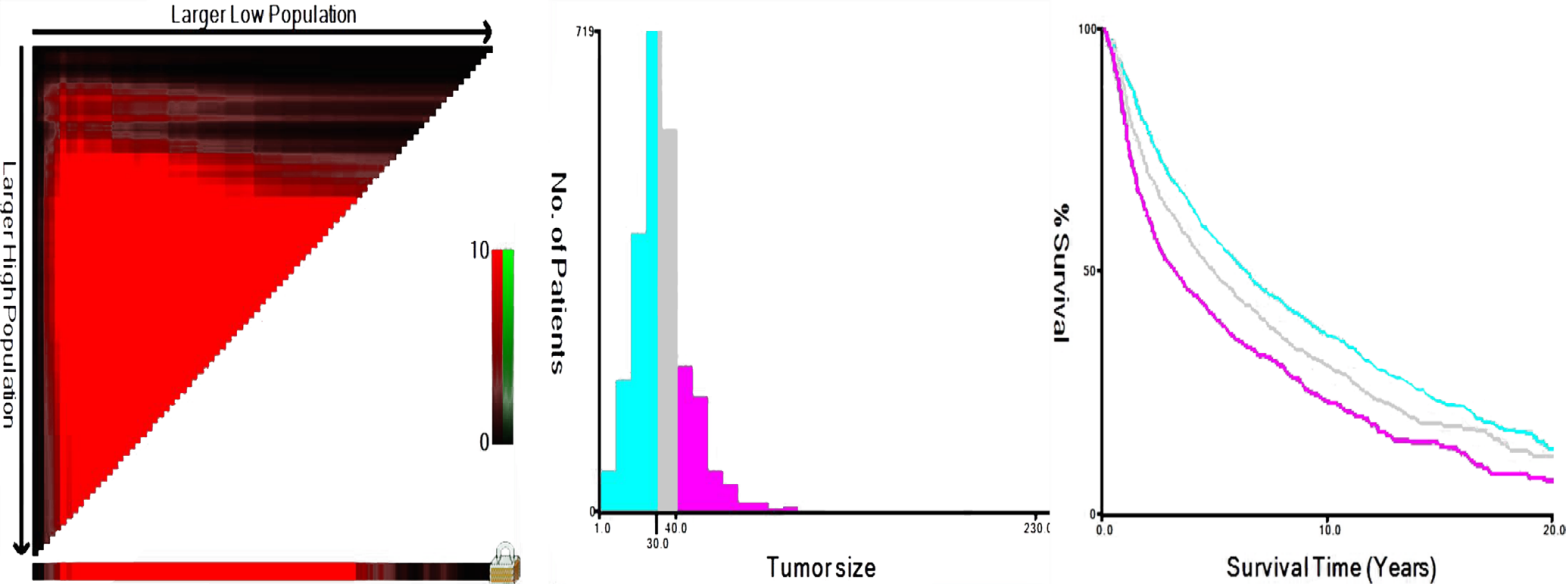
X-tile analysis identifying optimal tumor size cutoffs based on OS X-tile analysis was conducted on the training cohort of our study (*n* = 2477), these 2477 patients in the training cohort was equally divided into training (*n* = 1238) and validation sets (*n* = 1239). X-tile plots of training sets are shown in the left panels, the “lock” symbol in the left panel means optimal cutoffs have been determined, a histogram (middle panels) and a Kaplan-Meier plot (right panels) was performed based on these cutoffs. *P* values were determined by using the cut-point defined in the training set and applying it to the validation set. Optimal tumor size cut-points were identified as 30mm and 40mm based on OS (χ2 = 59.83, *P* < 0.001).

Just as the method of Imre’s and Ryu’s studies, when calculating cutoff values for LNR, we only included patients with positive lymph nodes (N+) [18, 19]. Based on overall survival, the program identified optimal LNR cutoff point for node-positive patients as 0.14, the optimal tumor size cutoffs for the entire training cohort (including both node-negative and node-positive patients) were 3.0 and 4.0 cm (Figures 2 and 3). While based on cancer-specific survival which was another primary endpoint of our interest, X-tile identified optimal LNR cut-point for node-positive patients as 0.12, while the optimal tumor size cutoffs for the entire cohort were 3.0 and 3.9 cm (Supplementary Figures 1 and 2). For the purpose of a unified standard, we adopted optimal cutoffs of LNR and tumor size based on OS, as X-tile utilized Kaplan-Meier method for analysis, which was more suitable for estimating overall survival.

**Figure 4:**
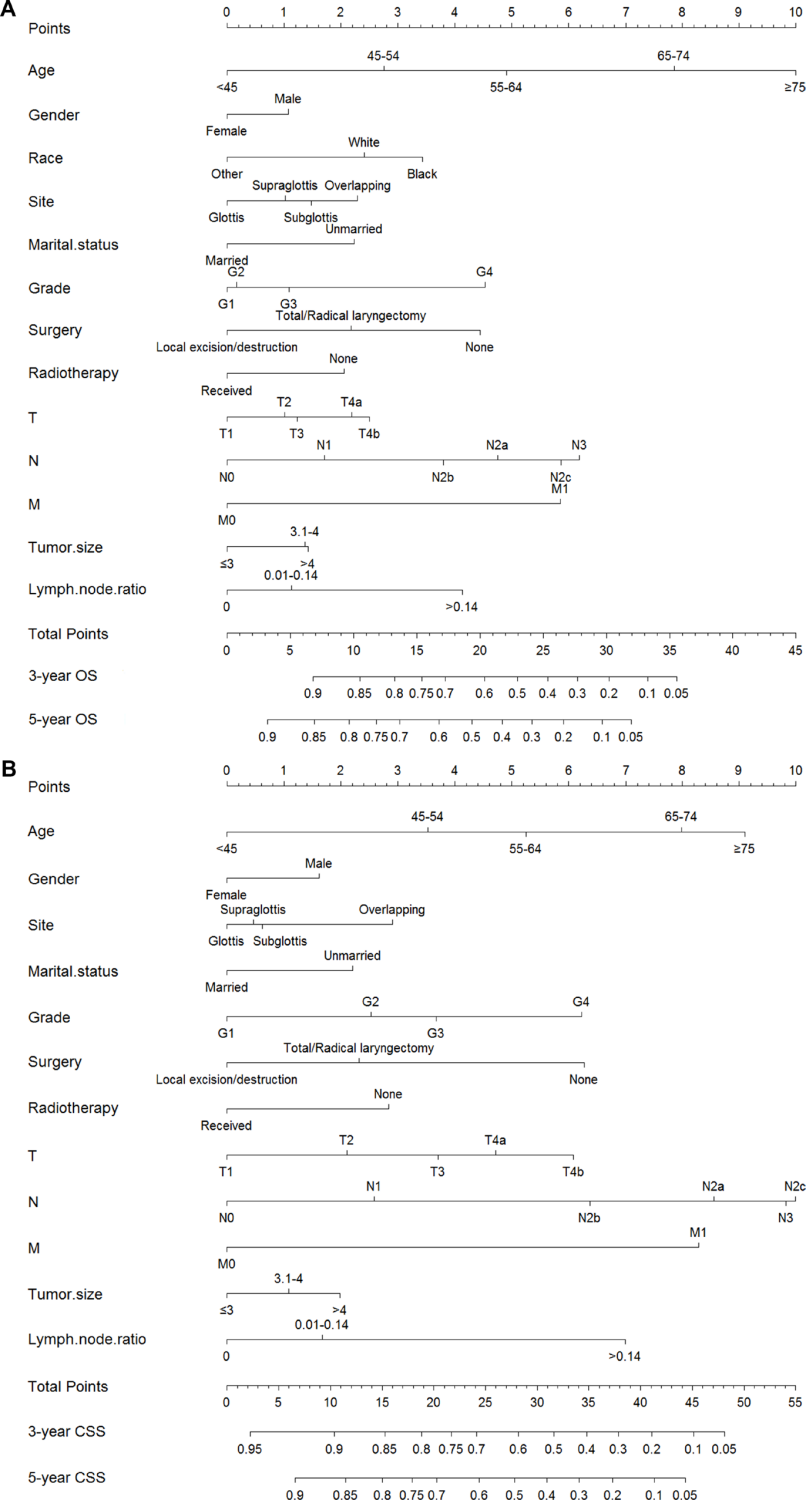
Nomograms estimating 3- and 5-year (**A**) overall survival (**B**) cancer-specific survival of LSCC patients treated with neck dissection. Instructions of the nomograms: First, each characteristic of an individual patient is located on the corresponding axis, we can draw a vertical line from that variable to the points scale to obtain its point (or look up Table 6). Second, we need to add up the points of each characteristic to obtain a total point, then draw a vertical line from the Total Points Scale to the 3- and 5-year OS or CSS scale to get the estimated probabilities of survival.

Consequently, the entire training cohort was divided into ≤ 3 cm, 3.1–4 cm and > 4 cm groups by tumor size. Those node-positive patients were divided into 0.01–0.14 group and > 0.14 group by LNR. When combined with node-negative patients, the entire training cohort was divided into LNR = 0, LNR 0.01–0.14 and LNR > 0.14 groups. Patients’ age at diagnosis was also stratified by ten-year age groups. Table 2 summarized baseline information on age, LNR and tumor size after categorization.

**Table 2:** Baseline information on age, tumor size and LNR after setting up optimal cutoff values

Characteristic	All patients		Training cohort		Validation cohort	
	*n* = 2752		*n* = 2477		*n* = 275	
	No.	%	No.	%	No.	%
**Age at diagnosis**						
< 45	190	6.9	171	6.9	19	6.9
45–54	617	22.4	560	22.6	57	20.7
55–64	1039	37.8	929	37.5	110	40.0
65–74	676	24.6	607	24.5	69	25.1
≥ 75	230	8.4	210	8.5	20	7.3
**Tumor size (cm)**						
≤ 3	1475	53.6	1320	53.3	155	56.4
3.1–4	701	25.5	637	25.7	64	23.3
> 4	576	20.9	520	21.0	56	20.4
**Lymph node ratio**						
0	1310	47.6	1175	47.4	135	49.1
0.01–0.14	889	32.3	805	32.5	84	30.5
> 0.14	553	20.1	497	20.1	56	20.4

SEER 1988–2008.

### Factors associated with OS in the training cohort

Of the 2477 patients in the training cohort, in the univariate analysis, all the demographics and tumor characteristics in Table 1 were associated with OS (*P* < 0.05) and no multicollinearity was observed among the variables (all VIFs < 5). These variables were included in the multivariate Cox proportional hazards regression model. Furtherly in the multivariate analysis, all the included characteristics remained significant for OS according to Wald test (*P* < 0.05) (Table 3).

**Table 3:** Univariate and multivariate analysis of overall survival in the training cohort

Characteristic	Univariate analysis	Multivariate analysis		
	*P* value	HR (95%CI)	*P*	*P* (Wald test)
**Age at diagnosis**	< 0.001			< 0.001
< 45		0.314 (0.244–0.405)	< 0.001	
45–54		0.433 (0.361–0.520)	< 0.001	
55–64		0.556 (0.470–0.657)	< 0.001	
65–74		0.781 (0.659–0.926)	0.004	
≥75		Reference		
**Gender**	< 0.001			0.038
Female		Reference		
Male		1.132 (1.007–1.272)	0.038	
**Race**	0.020			0.005
White		Reference		
Black		1.125 (1.005–1.259)	0.040	
Other		0.757 (0.597–0.959)	0.021	
**Marital status**	< 0.001			< 0.001
Married		Reference		
Unmarried		1.294 (1.176–1.423)	< 0.001	
**Grade**	< 0.001			0.043
Well differentiated		Reference		
Moderately differentiated		1.020 (0.861–1.208)	0.804	
Poorly differentiated		1.134 (1.037–1.249)	0.015	
Undifferentiated		1.685 (1.017–2.791)	0.043	
**Site**	0.011			0.040
Glottis		Reference		
Supraglottis		1.124 (0.936–1.351)	0.211	
Subglottis		1.186 (0.992–1.417)	0.056	
Overlapping lesion		1.301 (1.180–1.419)	< 0.001	
**Cancer-directed surgery**	< 0.001			< 0.001
No cancer-directed surgery		Reference		
Local excision/destruction		0.599 (0.444–0.808)	0.001	
Total/Radical laryngectomy		0.770 (0.581–1.020)	0.069	
**Radiotherapy**	0.005			< 0.001
No radiotherapy		Reference		
Receive radiotherapy		0.790 (0.706–0.884)	< 0.001	
**AJCC T status**	< 0.001			0.031
T1		Reference		
T2		1.124 (0.949–1.330)	0.175	
T3		1.154 (0.945–1.408)	0.161	
T4a		1.288 (1.083–1.531)	0.004	
T4b		1.338 (0.991–1.806)	0.057	
**AJCC N status**	< 0.001			< 0.001
N0		Reference		
N1		1.222 (0.562–2.649)	0.612	
N2a		1.732 (0.786–3.816)	0.173	
N2b		1.554 (0.721–3.352)	0.261	
N2c		1.969 (0.913–4.246)	0.084	
N3		2.044 (0.902–4.633)	0.087	
**AJCC M status**				< 0.001
M0		Reference		
M1		1.931 (1.364–2.734)	< 0.001	
**Tumor size (cm)**	< 0.001			0.005
≤ 3		Reference		
3.1–4		1.171 (1.046–1.310)	0.006	
> 4		1.177 (1.041–1.331)	0.009	
**Lymph node ratio**	< 0.001			< 0.001
0		Reference		
0.01–0.14		1.137 (0.530–2.441)	0.741	
> 0.14		1.606 (0.750–3.439)	0.223	

Abbreviations: HR: Hazard Ratio; CI: Confidence Interval.

^1^Including American Indian/Alaska Native, Asian/Pacific Islander.

^2^Including patients who are never married, divorced, separated or widowed.

^3^Local destruction includes photodynamic therapy, electrocautery, cryosurgery, laser ablation, etc.

(*n* = 2477).

### Cancer-specific mortality, competing risks and multivariate analysis for CSS

Cumulative incidence function (CIF), which was an unbiased way for analyzing cause-specific incidence when competing risk exists, was used to estimate cancer-specific mortality (CSM) and deaths resulting from other causes (DROC), as appropriate. The 3- and 5-year cumulative incidences of cancer-specific mortality (CICSM) were 30.1% and 37.2%, respectively, and the 3- and 5-year cumulative incidences of DROC were 6.2% and 11.3%, respectively. Gray’s test showed that all the variables, except race, proved to be associated with CSM (*P* < 0.05). We also observed that in patients of each subgroup except those ≥ 75 years old, CSM estimated by Kaplan-Meier method was quite close to the results by CIF, indicating that the low incidence of competing risks played a very slight interfering role in the Kaplan-Meier estimates for CSS (Table 4).

**Table 4:** 3- and 5-year cumulative incidences of death in the training cohort

Characteristic	Cumulative Incidence of CSM			CSM by Kaplan-Meier estimates		Cumulative Incidence of DROC		
	3-year	5-year	*P*	3-year	5-year	3-year	5-year	*P*
**All patients**	30.1%	37.2%		31.1%	39.5%	6.2%	11.3%	
**Age at diagnosis**			**< 0.001**					**< 0.001**
< 45	23.2%	29.4%		23.9%	30.4%	3.8%	6.8%	
45–54	27.6%	33.9%		28.1%	35.1%	3.4%	7.7%	
55–64	29.6%	36.2%		30.4%	37.9%	4.8%	9.1%	
65–74	32.6%	41.2%		34.1%	44.7%	8.7%	15.3%	
≥75	37.7%	46.3%		40.9%	52.9%	15.4%	23.0%	
**Gender**			**0.035**					**0.376**
Female	24.6%	33.9%		25.6%	36.3%	7.2%	9.8%	
Male	31.4%	38.0%		32.5%	40.2%	6.0%	11.7%	
**Race**			**0.160**					**0.258**
White	29.4%	36.4%		30.4%	38.5%	6.4%	11.4%	
Black	31.9%	40.0%		33.4%	42.8%	6.8%	11.9%	
Other	31.5%	37.2%		31.9%	38.0%	1.9%	6.5%	
**Marital status**			**0.008**					**0.046**
Married	26.5%	33.7%		27.2%	35.5%	5.5%	10.0%	
Unmarried	33.7%	40.8%		35.2%	43.6%	7.1%	12.8%	
**Grade**			**< 0.001**					**0.018**
Well differentiated	20.1%	24.8%		20.8%	26.3%	7.5%	14.4%	
Moderately differentiated	28.1%	35.6%		29.0%	37.6%	5.5%	10.2%	
Poorly differentiated	36.3%	43.6%		37.9%	46.7%	7.2%	12.2%	
Undifferentiated	40.4%	45.5%		40.8%	48.2%	7.3%	22.3%	
**Site**			**0.016**					**0.388**
Glottis	26.3%	32.9%		27.2%	34.8%	7.2%	11.4%	
Supraglottis	31.2%	38.5%		32.6%	41.2%	7.6%	12.0%	
Subglottis	33.8%	40.3%		33.9%	41.1%	3.6%	8.1%	
Overlapping lesion	36.6%	47.5%		36.5%	48.5%	1.4%	8.4%	
**Cancer-directed surgery**			**< 0.001**					**0.324**
No cancer-directed surgery	46.2%	57.9%		47.4%	62.3%	5.9%	11.6%	
Local excision/destruction	20.9%	28.2%		21.4%	29.5%	5.0%	8.3%	
Total/Radical laryngectomy	31.3%	38.3%		32.5%	40.7%	6.6%	11.9%	
**Radiotherapy**			**< 0.001**					**0.041**
No radiotherapy	26.8%	32.3%		28.1%	34.6%	7.9%	13.5%	
Receive radiotherapy	31.5%	39.4%		32.4%	41.5%	5.6%	10.4%	
**AJCC T status**			**< 0.001**					**0.071**
T1	21.0%	26.9%		21.6%	28.3%	6.0%	9.5%	
T2	26.1%	33.0%		27.5%	35.7%	8.3%	13.9%	
T3	24.4%	33.5%		24.7%	35.0%	4.2%	9.3%	
T4a	34.9%	41.8%		36.1%	44.0%	5.8%	11.2%	
T4b	53.7%	63.4%		56.2%	67.1%	4.7%	6.9%	
**AJCC N status**			**< 0.001**					**0.002**
N0	16.3%	23.3%		16.8%	24.2%	4.6%	10.0%	
N1	29.1%	35.9%		30.6%	38.8%	8.1%	14.6%	
N2a	42.2%	48.8%		46.4%	54.5%	11.2%	15.0%	
N2b	43.3%	51.2%		45.5%	54.9%	7.1%	11.4%	
N2c	55.7%	64.1%		59.5%	69.5%	7.1%	11.1%	
N3	51.7%	63.3%		54.5%	68.4%	8.9%	11.3%	
**AJCC M status**			**< 0.001**					**0.187**
M0	29.3%	36.5%		30.3%	38.7%	6.3%	11.4%	
M1	67.7%	71.6%		72.7%	77.3%	6.9%	6.9%	
**Tumor size (cm)**			**< 0.001**					**0.425**
≤ 3	24.8%	32.6%		22.5%	31.0%	5.3%	10.2%	
3.1–4	33.3%	39.9%		30.7%	39.3%	7.1%	13.1%	
> 4	39.5%	45.7%		41.5%	48.9%	7.9%	12.2%	
**Lymph node ratio**			**< 0.001**					**< 0.001**
0	16.5%	23.5%		17.0%	24.5%	4.6%	10.0%	
0.01–0.14	33.0%	41.4%		34.6%	44.0%	9.1%	14.2%	
> 0.14	54.9%	61.5%		57.2%	65.1%	5.9%	9.8%	

Abbreviations: CSM: cancer-specific mortality; DROC: death resulting from other causes.

P*: *P* value calculated by Gray’s test.

(*n* = 2477).

Owing to the limited interference effect of competing risks, Cox proportional hazards model rather than sub-distribution competing risks model was used to conduct multivariate analysis for CSS and build nomograms (Reasons for choosing Cox model was discussed furtherly in Discussion section). Furtherly, all the significant variables in the univariate test in Table 4 were confirmed to be independent prognostic factors for CSS in multivariate analysis (Table 5).

**Table 5:** Multivariate analysis of cancer-specific survival in the training cohort

Characteristic	Multivariate analysis		
	HR (95%CI)	*P* value	*P* (Wald test)
**Age at diagnosis**			**< 0.001**
< 45	0.448 (0.324–0.619)	< 0.001	
45–54	0.612 (0.485–0.772)	< 0.001	
55–64	0.712 (0.573–0.885)	0.002	
65–74	0.908 (0.726–1.134)	0.394	
≥75	Reference		
**Gender**			**0.046**
Female	Reference		
Male	1.152 (1.005–1.326)	0.046	
**Site**			**0.041**
Glottis	Reference		
Supraglottis	1.042 (0.829–1.309)	0.726	
Subglottis	1.058 (0.849–1.318)	0.617	
Overlapping lesion	1.291 (1.174–1.420)	< 0.001	
**Marital status**			**0.001**
Married	Reference		
Unmarried	1.214 (1.077–1.368)	0.001	
**Grade**			**0.036**
Well differentiated	Reference		
Moderately differentiated	1.253 (0.979–1.602)	0.073	
Poorly differentiated	1.385 (1.070–1.792)	0.013	
Undifferentiated	1.730 (0.862–3.474)	0.123	
**Cancer-directed surgery**			**0.005**
No cancer-directed surgery	Reference		
Local excision/destruction	0.576 (0.406–0.817)	0.002	
Total/Radical laryngectomy	0.706 (0.511–0.977)	0.035	
**Radiotherapy**			**0.001**
No radiotherapy	Reference		
Receive radiotherapy	0.780 (0.674–0.902)	0.001	
**AJCC T status**			**0.001**
T1	Reference		
T2	1.205 (0.958–1.515)	0.111	
T3	1.388 (1.068–1.804)	0.014	
T4a	1.517 (1.202–1.914)	< 0.001	
T4b	1.713 (1.191–2.466)	0.004	
**AJCC N status**			**< 0.001**
N0	Reference		
N1	1.259 (0.502–3.155)	0.623	
N2a	2.128 (0.837–5.409)	0.113	
N2b	1.757 (0.706–4.373)	0.226	
N2c	2.414 (0.970–6.004)	0.058	
N3	2.375 (0.903–6.250)	0.081	
**AJCC M status**			**< 0.001**
M0	Reference		
M1	2.054 (1.389–3.038)	< 0.001	
**Tumor size (cm)**			**0.045**
≤ 3	Reference		
3.1–4	1.099 (0.952–1.270)	0.198	
> 4	1.191 (1.023–1.386)	0.025	
Lymph node ratio			**< 0.001**
0	Reference		
0.01–0.14	1.159 (0.469–2.862)	0.749	
> 0.14	1.850 (0.751–4.555)	0.181	

Abbreviations: HR: Hazard Ratio; CI: Confidence Interval. (*n* = 2477).

### Establishment and validation of the nomograms

By a backward stepwise method based on the smallest Akaike Information Criterion (AIC), all the significant factors in multivariate analysis were incorporated to develop nomograms predicting 3- and 5-year OS and CSS (Figure 4). Detailed scores of each nomogram predictor were presented in Table 6.

**Figure 5:**
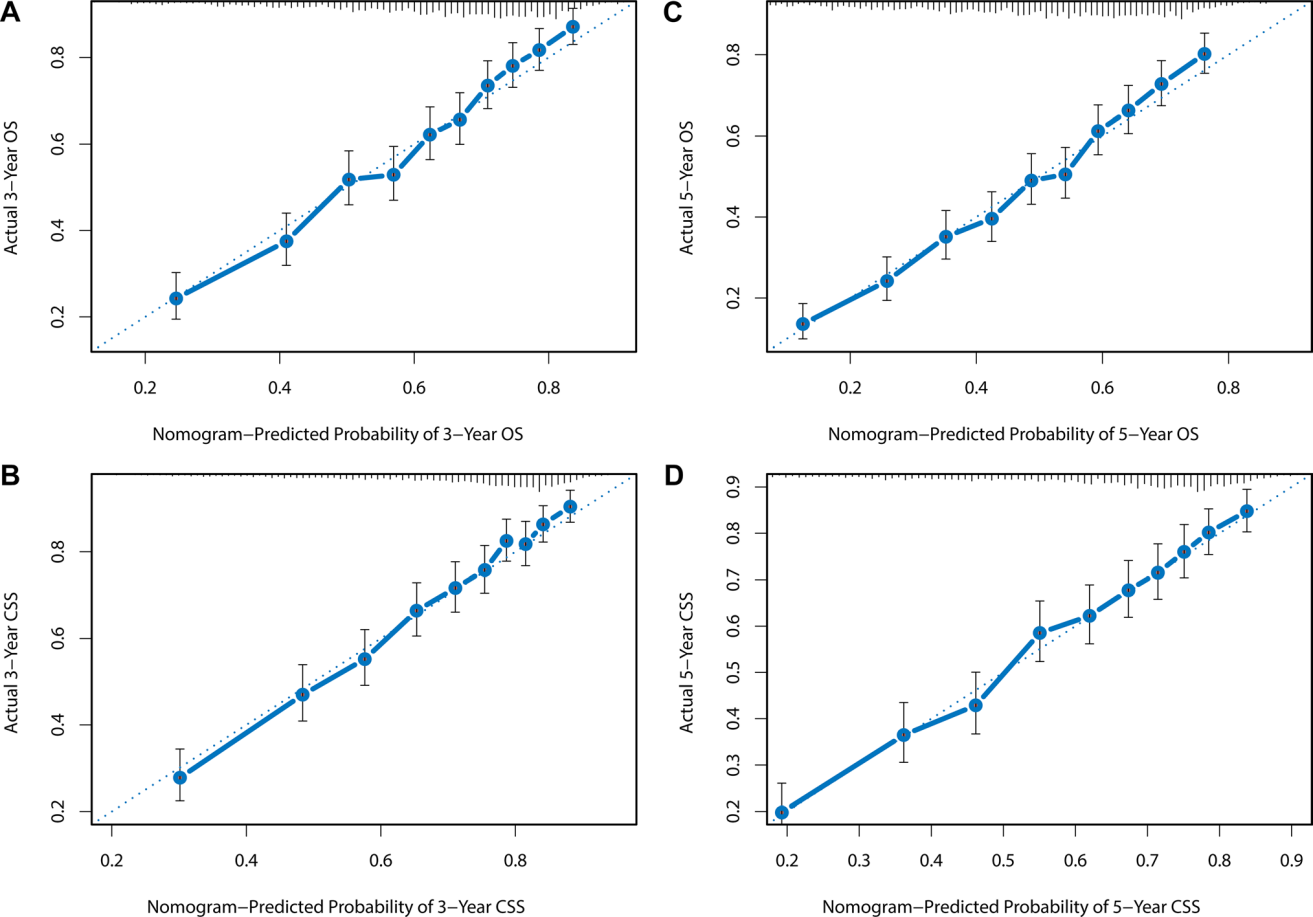
Internal calibration plots for (**A**) 3-year OS, (**B**) 5-year OS, (**C**) 3-year CSS, (**D**) 5-year CSS. The diagonal dashed line in each plot represents perfect match between nomogram prediction (x-axis) and actual observed survival (y-axis). The training cohort was divided into 10 groups with equal sample size for internal validation. Closer distances between the fit line and the diagonal line indicate higher prediction accuracy.

**Table 6:** Detailed scores of each predictor in the nomograms

Characteristic	OS nomogram	CSS nomogram
**Age at diagnosis**		
< 45	0.0	0.0
45–54	2.8	3.5
55–64	4.9	5.3
65–74	7.9	8.0
≥ 75	10.0	9.1
**Gender**		
Female	0.0	0.0
Male	1.1	1.6
**Race**		
White	2.4	
Black	3.4	Not included
Other	0.0	
**Marital status**		
Married	0.0	0.0
Unmarried	2.2	2.2
**Grade**		
Well differentiated	0.0	0.0
Moderately differentiated	0.2	2.5
Poorly differentiated	1.1	3.7
Undifferentiated	4.5	6.2
**Site**		
Glottis	0.0	0.0
Supraglottis	1.0	0.5
Subglottis	1.5	0.6
Overlapping lesion	2.3	2.9
**Cancer-directed surgery**		
No cancer-directed surgery	4.4	6.3
Local excision/destruction	0.0	0.0
Total/Radical laryngectomy	2.2	2.3
**Radiotherapy**		
No radiotherapy	2.1	2.8
Receive radiotherapy	0.0	0.0
**AJCC T status**		
T1	0.0	0.0
T2	1.0	2.1
T3	1.2	3.7
T4a	2.2	4.7
T4b	2.5	6.1
**AJCC N status**		
N0	0.0	0.0
N1	1.7	2.6
N2a	4.8	8.6
N2b	3.8	6.4
N2c	5.9	10.0
N3	6.2	9.8
**AJCC M status**		
M0	0.0	0.0
M1	5.9	8.3
**Tumor size (cm)**		
≤ 3	0.0	0.0
3.1–4	1.4	1.1
> 4	1.5	2.0
**Lymph node ratio**		
0	0.0	0.0
0.01–0.14	1.1	1.7
> 0.14	4.1	7.0

**Figure 6:**
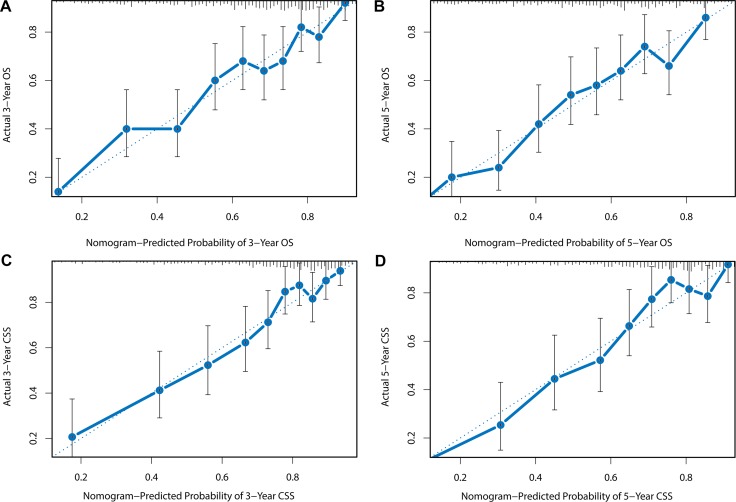
External calibration plots for (**A**) 3-year OS, (**B**) 5-year OS, (**C**) 3-year CSS, (**D**) 5-year CSS. The diagonal dashed line in each plot represents perfect match between nomogram prediction (X-axis) and actual observed survival (Y-axis). The validation cohort was divided into 10 groups with equal sample size for external validation. Closer distances between the fit line and the diagonal line indicate higher prediction accuracy.

The nomograms were validated by bootstrap resampling internally and externally. Harrell’s Concordance-indexes (C-index), as an indicator of predictive abilities, were compared among nomogram models, no-LNR models (including all the variables in the nomograms except LNR) and TNM models (including only TNM status). As shown in Table 7, in the internal validation via training cohort, the nomogram models possessed higher C-indexes (OS: 0.713; CSS: 0.725) than no-LNR model (OS: 0.703; CSS: 0.713) and TNM model (OS: 0.667; CSS: 0.688). In the validation cohort for external validation, similarly, nomogram model (OS: 0.704; CSS: 0.709) still demonstrated superiority over no-LNR model (OS: 0.690; CSS: 0.693) and TNM model (OS: 0.658; CSS: 0.672). C-index and AIC of each model were presented in Table 7. Internal and external calibration plots for OS and CSS also showed good agreement between nomogram prediction and observed outcomes (Figures 5 and 6).

**Table 7:** Predictive ability of different prediction models

Predicting models		Harrell’s C-index	AIC
**Internal validation**	**Overall survival**		
	TNM classification	0.667	26193.53
	No-LNR model	0.703	25971.86
	Nomogram model	0.713	25946.89
	**Cancer-specific survival**		
	TNM classification	0.688	16491.48
	No-LNR model	0.713	16419.76
	Nomogram model	0.725	16385.39
**External validation**	**Overall survival**		
	TNM classification	0.658	6096.82
	No-LNR model	0.690	6075.84
	Nomogram model	0.704	6052.44
	**Cancer-specific survival**		
	TNM classification	0.672	3948.56
	No-LNR model	0.693	3947.39
	Nomogram model	0.709	3921.78

Abbreviations: AIC: Akaike Information Criterion.

SEER 1988–2008.

It’s easy and comprehensible to predict survival by nomograms which possess a user-friendly interface. Based on an individual patient’s information, we can look up Table 6 to obtain the point of each prognostic factor, and then add up the points and predict survival by the corresponding survival probabilities of the total points. Take this example, for a married 60 year-old white man, he was diagnosed with well-differentiated T4aN1M0 glottis squamous cell carcinoma with a primary tumor of 2.5 cm in greatest diameter, he didn’t undergo cancer-directed surgery but received radiotherapy, neck dissection showed 5% of examined lymph nodes had signs of metastasis. In summary, he got 17.8 and 22.2 points in OS and CSS nomograms, respectively, corresponding to an estimated 56% 5-year OS rate and 65% 5-year CSS rate. In the same manner, we could also predict a relatively short-term 3-year survival rate with the nomograms.

## DISCUSSION

Cancer prognosis has proved to be closely related to different aspects of factors, such as demographics, tumor characteristics and treatment conditions. AJCC TNM status, which has been the most widely used system for outcome estimation, however, is not sufficient to satisfy current need. Nomogram, considered to be a graphical depiction of prediction model, gives oncologists an opportunity to combine different tumor prognostic factors together, thus to help us assess the risk of failure more precisely [20, 21].

Regardless of surgery or radiotherapy as treatment for primary tumor, neck dissection is recommended for LSCC patients with potential node involvement [7]. LNR information obtained from neck dissection could also help improve our understanding of node metastasis in depth. Published nomograms developed in Ju’s and Shen’s studies focused on prognosis of squamous cell carcinoma of all sites of head and neck based on SEER database with large sample size, however, patients included in their studies didn’t necessarily need to receive neck dissection thus information on LNR of these patients was not available to be incorporated into the nomograms. Moreover, as characteristics of tumors from different sites of head and neck may vary greatly, it might not be most appropriate to use the same predictive tool to estimate prognosis of different head and neck cancer. When we predicted survival of LSCC patients treated with neck dissection, for these reasons above, the nomograms in Ju’s and Shen’s researches were not able to achieve the best predictive performance [22, 23]. Consequently, in terms of necessity, we established more comprehensive nomograms to predict survival of LSCC patients who underwent neck dissection and they proved to be more accurate than previous models as well as conventional TNM staging systems. Since SEER program is comprised of 18 cancer registries covering thousands of hospitals and nearly 30% of total population across the nation, heterogeneity of the data allowed the models to be broadly used for decision-making in clinical practice.

Through univariate Log-rank test and multivariate Cox analysis, independent predictive factors for OS were identified. When we selected the variables to build nomograms, backward stepwise methods were used to determine the smallest AIC value in order to minimize the information loss. With regard to CSS, according to Fine and Gray, an overestimation of failure is expected due to the existing competing risks when Kaplan-Meier and Cox proportional hazards analysis were applied [24]. However, in the comparison between Kaplan-Meier estimates and the CIF means, very close CSM results suggested the relative low incidence of competing risks in our study might not be a critical consideration. In view of Cox models’ high interpretability by nomogram and comparability with previous literatures, just as the method of Valentini’s research and some other prior studies [25–27], we still adopted a multivariate Cox proportional hazards model for CSS analysis.

In our study, we validated some clinicopathological factors as important prognostic indicators for both OS and CSS in the multivariate analysis, including sociodemographic factors like age at diagnosis, gender, marital status; tumor characteristics such as primary site, grade, TNM status, tumor size and lymph node ratio; as well as treatment conditions like surgery and radiotherapy. As shown in our nomograms, age at diagnosis revealed strong impact on OS and CSS. In average, compared with a patient less than 45 years old, 5-year OS and CSS rates were reduced by 35% and 20% for those over 75, even if they had the same exposure to other risk factors. Age was also proved to be an important predictive and prognostic factor in some previous studies. Ampil et al confirmed that age was a prognostic factor in T4 laryngeal carcinoma [28], Kowalski et al also proved age as an important prognostic factor in T3N0-1 glottic or transglottic cancer [29]. In another study of 945 cases in Sweden, Reizenstein et al reported that elderly laryngeal cancer patients received higher proportion of palliative care rather than radical treatment and thus to have a much higher never-free-from-tumor rate, which might be a potential reason for their increased risk of mortality [30]. In our study, male patients were found to have a significantly greater risk of both overall and cancer-specific mortality compared with female. It has been reported by Shan SJ et al that male head and neck cancer patients were relatively more reluctant to undergo follow-up screening than female [31]. Besides, according to Sharp et al ’s research based on a large national cancer database in Ireland, smoking at diagnosis is an independent prognostic factor for cancer-specific survival in head and neck cancer and proportion of current smokers at diagnosis in male patients was much higher than in female [32]. These reasons could partly explain why gender was an independent prognostic and predictive factor for cancer-specific survival. According to our research, marriage demonstrated a significant protective effect, relationship between marital status and cancer outcomes was also certified in many cancer types [33–36], the role of spousal support in behavior change, psychological regulation and treatment compliance might be potential mechanisms [37, 38]. Previous literatures reported that African American people were generally in a lower social class, and possessed worse socioeconomic conditions and living habits, thus to have a higher risk for comorbidities, this could reasonably explain why race was only statistically significantly associated with OS, but not CSS (*P* = 0.160) in the univariate analysis of our study [39–43]. It’s also noteworthy that in the CSS nomogram, N staging had the greatest effect on CSS and in particular, LNR made an even bigger contribution than T categories to both OS and CSS, indicating that nodal status had a stronger influence on prognosis than status of primary tumor, which was in consistence with the opinion by Ferlito et al [5].

Inevitably, potential limitations in our study should be taken into consideration. First, important therapies like chemotherapy were not accessible in SEER database and SEER no longer recorded the type or regions of neck dissection after 2003, besides, extracapsular spread (ECS) as an important prognostic factor for laryngeal cancer patients with positive lymph nodes, wasn’t recorded in SEER database as well. Consequently these factors couldn’t be recruited in the nomograms. Second, during the 20 years between 1988 and 2008, compared to all the LSCC patients registered in SEER database, those who underwent neck dissection were relatively small (less than 3000), causing a lack of patients in some subgroups, like there were only 20 patients in undifferentiated grade subgroup, which probably reduced accuracy. Third, SEER didn’t collect information on some important common-recognized laboratory prognostic indices like SCC-Ag expression, EGFR or VEGF mutation [44, 45], they could furtherly improve the predictive performance if incorporated. Fourth, in addition to LNR, positive nodes count and log odds of positive lymph nodes (LODDS) were also potential prognostic indicators provided by neck dissection, further comparisons of their prognostic capabilities were required. Moreover, the nomograms should be validated by prospective research on account of the retrospective nature of our study.

In conclusion, we constructed and validated nomograms estimating overall and cancer-specific survival of laryngeal squamous cell carcinoma patients treated with neck dissection using a large, population-based dataset. These nomograms exhibited high degree of applicability and accuracy, outperforming predictive models in previous literatures as well as TNM staging system. By these predictive tools, it will be more effective to assist clinicians in identifying patients with high risk of mortality and making more precise survival evaluation.

## MATERIALS AND METHODS

### Data source and inclusion criteria

All the data was obtained from Surveillance, Epidemiology, and End Results (SEER) database, which collects information of cancer patients in 18 registries, covering approximately 30% of total U.S population. The database includes some important demographic, diagnostic and treatment information of cancer patients, such as primary sites, morphology, stage, surgery, radiotherapy, grade and patients’ vital status. (http://www.seer.cancer.gov) SEER*Stat software (Version 8.3.2) was used to extract information from the database.

The inclusion criteria were as follows: 1) Diagnosed with LSCC as its first and only malignancy. 2) The ICD-O-3 site codes were limited to C32.0 (glottis), C32.1 (supraglottis), C32.2 (subglottis), and C32.8 (overlapping lesion) according to SEER classification. It’s worth noting that C32.3 (Laryngeal cartilage) and C32.9 (NOS) were not included because the number of eligible patients was too small (less than 10). 3) Histological type was limited to squamous cell carcinoma (8052, 8070-8078, 8083-8084, 8094, 8560) according to ICD-O-3 histological codes, as appropriate. 4) Underwent neck dissection with definite counts of examined and positive lymph nodes. 5) Diagnosed between 1988 and 2008 to ensure an adequate follow-up length, as the follow-up cutoff date of currently available SEER data was 12/31/2013. 6) Known survival months after diagnosis and known cause of death. 7) Older than 18 years old. 8) Definite information on race, TNM status, grade, surgery, radiotherapy and tumor size. 9) Active follow-up. 10) Excluded if the diagnosis was obtained by death certificate or autopsy only.

### Statistical analysis for OS and CSS

The entire group of enrolled patients (*n* = 2752) was randomly divided into a training cohort (*n* = 2477) and a validation cohort (*n* = 275) in order to develop and validate nomograms. Patients’ 13 important clinicopathological factors including age at diagnosis, race, gender, marital status, site, grade, TNM status, surgery, radiotherapy, tumor size and lymph node ratio were used to conduct the univariate and multivariate analysis. Optimal cutoffs of tumor size and LNR were determined by X-tile program. Age at diagnosis was grouped by ten years, we combined patients less than 45 years old into one single group because only 15 patients were less than 35, similarly, those older than 75 years old were put into the same group as only 10 individuals in the training cohort were beyond 85.

One of our primary endpoints of interest was OS, defined as the time from diagnosis to death from all possible causes, patients who were alive at the time of last follow-up were counted as censored observations. Kaplan-Meier analysis and log-rank tests were used to identify the significant factors associated with OS. Multivariate Cox proportional hazards models were constructed to analyze the independent effect of significant factors in the univariate log-rank test and present the hazard ratios of different variables.

Another primary endpoint of our interest was CSS, measured as the time from diagnosis to death attributed to LSCC, patients who were alive at the time of last follow-up were counted as censored observations. Cumulative incidence function (CIF) was used to evaluate cancer-specific mortality (CSM) and death resulting from other causes (DROC) which was regarded as competing risks. CIF results of CSM in each subgroup were compared with those calculated by Kaplan-Meier estimates, a Cox regression model was used to conduct multivariate analysis and build a nomogram as it was easy to interpret, compare and comprehend [25, 26].

### Construction and validation of the nomograms

Nomograms were constructed based on Cox proportional hazards regression model to predict 3- and 5-year OS and CSS. To minimize the information loss, a backward stepwise method was used to recruit the independent prognostic factors into the construction of the nomograms until the minimal AIC value occurred.

Via the training cohort and the validation cohort, both internal and external nomogram validations were conducted. Performance of these predictive models was assessed by Harrell’s concordance-index (C-index), which was similar to area under curve (AUC), but proved to be more suitable for censored data [46]. C-index ranges from 0.5 to 1.0, 0.5 means total chance while 1.0 stands for prefect matching [12]. We also assessed the predictive performance by calibration plot, which was quantified by the comparison between nomogram-predicted survival with observed survival. Bootstraps with 1000 resample were used to conduct these activities [14].

Kaplan-Meier estimates, multivariate Cox regression and log-rank test were analyzed using statistical software IBM SPSS, version 22 (SPSS Inc, Chicago, IL, USA). Cumulative incidence function (CIF), Gray’s test, nomogram construction, validation and calibration were performed in R version 3.3.1 (http://www.r-project.org/) with *rms* [47] and *cmprsk* [48] packages. All *P* values were two-sided and statistical significance was set at *P* < 0.05.

### Ethics statement

Our study was approved by Shanghai Cancer Center Ethical Committee. We do not need informed patient consent for the data released by the SEER database.

## SUPPLEMENTARY MATERIALS FIGURES


