# Supplementary material for: Development of comprehensive nomograms for evaluating overall and cancer-specific survival of laryngeal squamous cell carcinoma patients treated with neck dissection
Source: Oncotarget. 2017 Feb 16;8(18):29722–40. doi: 10.18632/oncotarget.15414 (PMC5444698; doi:10.18632/oncotarget.15414)
Supplement: Supplementary file 1 [file oncotarget-08-29722-s001.pdf]

Development of comprehensive nomograms for evaluating overall and cancer-specific survival of laryngeal squamous cell carcinoma patients treated with neck dissection

Supplementary Materials

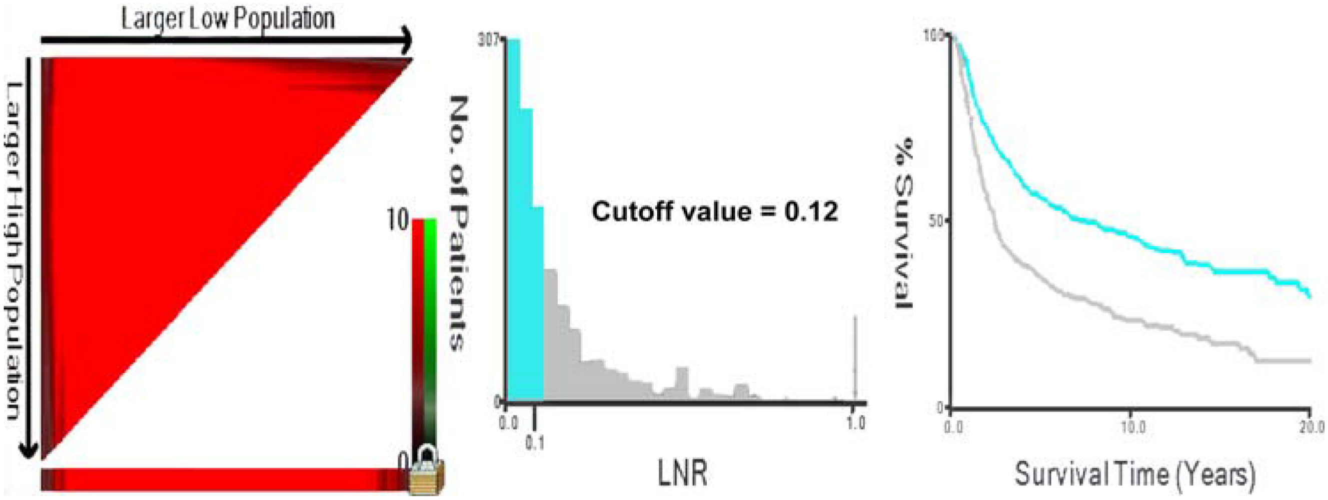

Supplementary Figure 1: X-tile analysis identifying optimal LNR cutoffs based on CSS. X-tile analysis was conducted on patients with positive lymph nodes in the training cohort (*n* = 1312), these 1312 patients in the training cohort was equally divided into training (*n* = 656) and validation sets (*n* = 656). X-tile plots of training sets are shown in the left panels, the "lock" symbol in the left panel means optimal cutoffs have been determined, a histogram (middle panels) and a Kaplan-Meier plot (right panels) was performed based on these cutoffs. P values were determined by using the cut-point defined in the training set and applying it to the validation set. Optimal LNR cut-point was determined as 0.12 based on CSS ($\chi2 = 74.499$, $P < 0.001$). As the X axis could only show one decimal place, so we added a text annotation "Cutoff value = 0.12" in the middle panel.

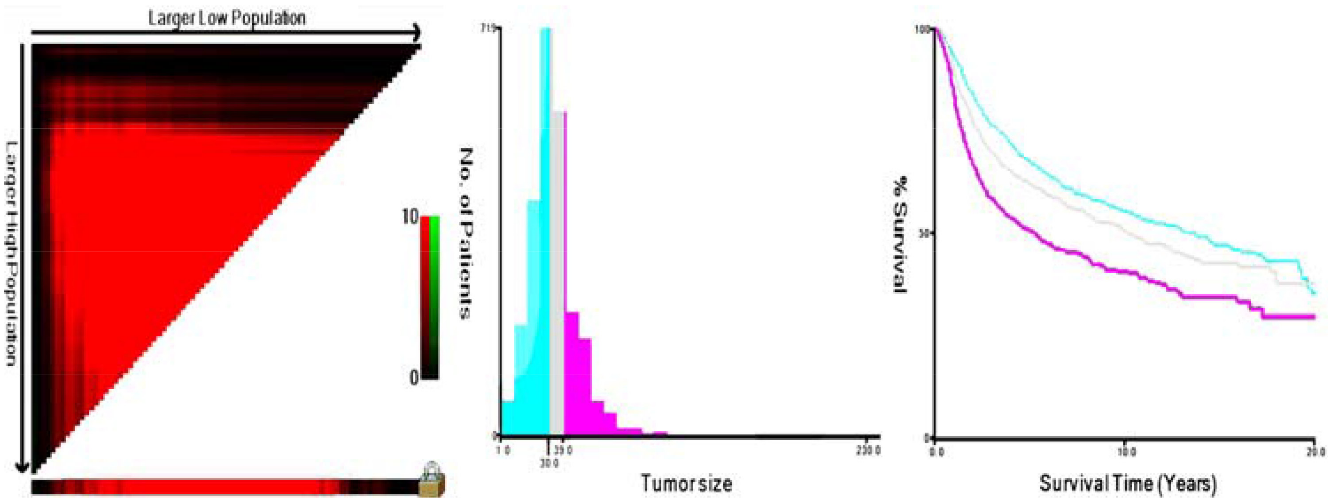

Supplementary Figure 2: X-tile analysis identifying optimal tumor size cutoffs based on CSS. X-tile analysis was conducted on the training cohort of our study (*n* = 2477), these 2477 patients in the training cohort was equally divided into training (*n* = 1238) and validation sets (*n* = 1239). X-tile plots of training sets are shown in the left panels, the "lock" symbol in the left panel means optimal cutoffs have been determined, a histogram (middle panels) and a Kaplan-Meier plot (right panels) was performed based on these cutoffs. P values were determined by using the cut-point defined in the training set and applying it to the validation set. Optimal tumor size cut-points were identified as 30 mm and 39 mm based on CSS ($\chi2 = 55.34$, $P < 0.001$).
